# Afatinib + bevacizumab combination therapy in *EGFR*‐mutant NSCLC patients with osimertinib resistance: Protocol of an open‐label, phase II, multicenter, single‐arm trial

**DOI:** 10.1111/1759-7714.13503

**Published:** 2020-06-03

**Authors:** Nobuaki Kobayashi, Hisashi Hashimoto, Chisato Kamimaki, Ryo Nagasawa, Katsushi Tanaka, Sousuke Kubo, Seigo Katakura, Hao Chen, Nobuyuki Hirama, Ryota Ushio, Ayako Aoki, Kentaro Nakashima, Shuhei Teranishi, Saki Manabe, Hiroki Watanabe, Nobuyuki Horita, Keisuke Watanabe, Yu Hara, Masaki Yamamoto, Makoto Kudo, Hongmei Piao, Takeshi Kaneko

**Affiliations:** ^1^ Department of Pulmonology Yokohama City University Graduate School of Medicine Yokohama Japan; ^2^ Respiratory Disease Center Yokohama City University Medical Center Yokohama Japan; ^3^ Department of Respiratory Medicine Affiliated Hospital of Yanbian University Yanji China

**Keywords:** Afatinib, bevacizumab, EGFR, non‐small cell lung cancer, osimertinib

## Abstract

**Introduction:**

As most patients with epidermal growth factor receptor (*EGFR*)‐mutant non‐small cell lung cancer (NSCLC) develop progressive disease after treatment with osimertinib, it is important to develop more effective treatment options. Afatinib has been shown to be more effective in in vitro studies than osimertinib when used in cancer cell lines containing some specific *EGFR* mutations. Therefore, afatinib may be an effective solution, especially when used in combination with an anti‐VEGF agent such as bevacizumab.

**Methods:**

A phase II multicenter, open‐label, single‐arm trial has been initiated to evaluate the efficacy and safety of afatinib and bevacizumab combination as salvage therapy for *EGFR*‐mutated lung cancer in patients previously treated with osimertinib. The primary endpoint will be the objective response rate (ORR) and secondary endpoints are progression‐free survival (PFS), overall survival (OS), disease control rate (DCR), and adverse events (AEs).

**Discussion:**

A previous study indicated that afatinib inhibits lung cancer cells with specific *EGFR* mutations more effectively than other EGFR‐TKIs such as osimertinib. Therefore, we expect that combination therapy using afatinib and bevacizumab will be effective in patients previously treated with osimertinib (registration no. jRCTs031190077).

## Introduction

Lung cancer‐associated deaths rank at the top of site‐specific statistics for malignant tumors worldwide. Nonsquamous non‐small cell lung cancers (NSCLC) account for approximately 65% of lung cancers in men and 85% of lung cancers in women; 30%–40% of these have mutations in the epidermal growth factor receptor (EGFR) gene in patients in Asia.^1^ Due to the high incidence of *EGFR* mutation‐positive lung cancers, further investigation into available treatment options is crucial.

In the phase III FLAURA trial, a randomized, double‐blinded trial comparing osimertinib with first‐generation EGFR‐tyrosine kinase inhibitors (EGFR‐TKIs), the median progression‐free survival (PFS) in patients with *EGFR*‐mutated (exon 19 deletion or L858R) lung cancer was significantly longer when treated with osimertinib than when treated with first‐generation EGFR‐TKIs (18.9 months vs. 10.2 months, respectively). Moreover, patients treated with osimertinib experienced fewer severe adverse events (AEs) than those treated with first‐generation EGFR‐TKIs.^2^ Based on these results, a large majority of patients with advanced *EGFR* mutation‐positive lung cancers received osimertinib as the first‐line therapy. Although recent improvements have been made in first‐line therapy, better salvage treatment for patients who have developed resistance to osimertinib is needed. Currently, the treatment for these patients is similar to that for those without the driver oncogene mutation, that is, systemic combination chemotherapy with cytotoxic drugs and/or immune checkpoint inhibitors. The treatment after progression of salvage chemotherapy is the same as that given for patients without driver oncogene mutations.

Several mechanisms for osimertinib resistance such as *EGFR* mutation C797S, loss of T790M, transformation to small cell lung cancer, MET/HER2 amplification, activation of the RAS‐mitogen‐activated protein kinase (MAPK), and RAS‐phosphatidylinositol 3‐kinase (PI3K) pathways have been identified.^3^, ^4^ Moreover, it is possible that compound *EGFR* mutations might play a role in the osimertinib resistance mechanism.^5^ Afatinib is thought to be effective for treating resistant cancers containing minor and compound *EGFR* mutations and HER2 amplification after osimertinib treatment. Moreover, Kohsaka *et al*. reported that the half maximal inhibitory concentration (IC_50_) of afatinib was more likely to inhibit the growth of cancer cell lines that have undergone in vitro mutagenesis.^6^, ^7^

Antivascular endothelial growth factor (VEGF) therapy inhibits tumor neovascularization, which is essential for tumor growth, and modifies the tumor microenvironment. Bevacizumab, a recombinant monoclonal antibody targeting VEGF‐A, is one of the most widely available anti‐VEGF therapies and has demonstrated a synergistic effect with EGFR‐TKIs against *EGFR* mutation‐positive lung cancers.^8^, ^9^

We thus conducted a phase II multicenter, open‐label, single‐arm trial to evaluate the efficacy and safety of afatinib and bevacizumab combination therapy for *EGFR* gene mutant lung cancers previously treated with osimertinib.

## Methods

### Study design and objectives

**Figure 1:**
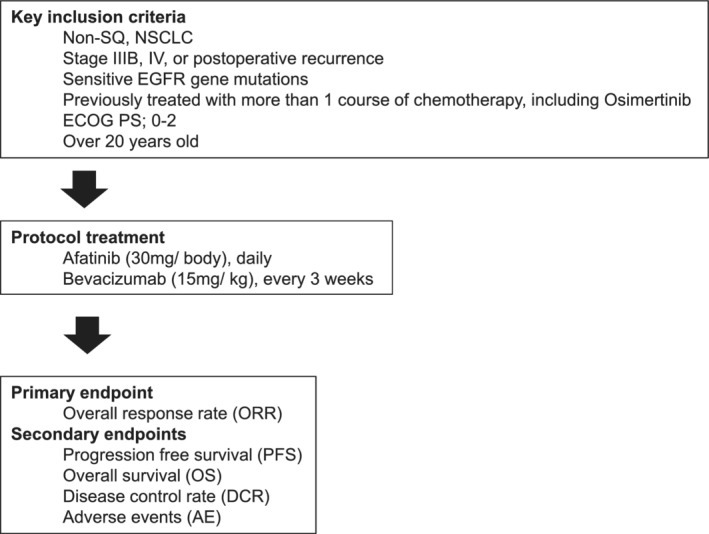
Study schema.

This study is designed as a phase II multicenter, open‐label, single‐arm trial to evaluate the efficacy and safety of afatinib and bevacizumab combination therapy in patients with *EGFR* gene mutated lung cancers previously treated with osimertinib. A total of 37 patients have been enrolled and treated with the study regimen (afatinib and bevacizumab). The primary endpoint is the objective response rate (ORR). The secondary endpoints are PFS, overall survival (OS), disease control rate (DCR), and AEs. The trial will be conducted in accordance with the principles of the Declaration of Helsinki and the International Conference on Harmonization Good Clinical Practice Guidelines, local laws, and regulations. This study was approved by the Yokohama City University Clinical Research Review Board (registration no. jRCTs031190077) (Fig 1).

#### Patient enrollment

All study participants are confirmed to meet the inclusion criteria, and each participant has provided informed consent. All patients who do not meet the inclusion criteria have been excluded. The inclusion and exclusion criteria are shown in Table 1.

**Table 1:** Inclusion and exclusion criteria

**Inclusion criteria**
Histologically‐ or cytologically‐confirmed nonsquamous, non‐small cell lung cancer **(NSCLC)**
Stage IIIB, IV, or postoperative recurrence
Sensitive *EGFR* gene mutations
Measurable disease based on RECIST guidelines 1.1.
Loss of response or intolerance to first‐line therapy
More than one course of chemotherapy, including osimertinib
Afatinib naïve
Eligible to receive adjuvant chemotherapy
Patients treated with radiotherapy are eligible if they meet the following criteria: target lesions are not involved in the radiation field; longer than 12 weeks since the last palliative radiation exposure to chest bone lesions; longer than two weeks since last irradiation treatment to areas other than the chest at the time of registration
At the time of registration, the following time periods have passed following the treatment: operation ‐ four weeks or more; continuous chest drainage ‐ two weeks or more; pleural adherence without antineoplastic agents ‐ two weeks or more
ECOG PS; 0, 1, 2
Minimum expected survival: three months
Baseline organ function and laboratory values that meet the following criteria: WBC ≥1500/mm^3^; neutrophils ≥1000/mm^3^; Hb ≥8.0 g/dL without blood transfusion within 14 days before registration; PLT ≥10 times 104/mm^3^; TBil ≤1.5 mg/dL; AST ≤100 U/L; ALT ≤100 U/L; Plasma creatinine ≤1.5 mg/dL; SpO2 ≥93%
Written informed consent is provided
Over 20 years old
Both males and females
**Exclusion criteria**
Interstitial pneumonia or pulmonary fibrosis
Multiple cancers
Pleural effusion, ascites, and pericardial effusion requiring pericardiocentesis
Cases with the following serious complications; uncontrolled angina, myocardial infarction, and heart failure within the previous three months; uncontrollable diabetes or hypertension; uncontrollable proteinuria; severe infection; severe diarrhea; hemoptysis (over 2.5 mL of fresh blood); other severe complications (eg ileus, superior vena cava syndrome, etc)
Nursing or pregnant women
Any patient deemed inappropriate by the attending physician

ALT, alanine aminotransferase; AST, aspartate transaminase; ECOG, Eastern Cooperative Oncology Group; EGFR, epidermal growth factor receptor; Hb, hemoglobin; PS, performance status; PLT, platelets; RECIST, response evaluation criteria in solid tumors; WBC, white blood cell; TBil, total bilirubin.

### Treatments

The enrolled patients will be treated with afatinib and bevacizumab combination therapy. Afatinib (30 mg) will be administered once daily orally, beginning on day one of cycle one, and bevacizumab (15 mg/kg) will be administered every three weeks intravenously on day one. Treatment will be continued until treatment discontinuation criteria are met.

### Efficacy and safety assessments

Computed tomography (CT) or positron emission tomography‐computed tomography (PET‐CT) will be performed once every eight weeks for six months from the initiation of treatment and once every nine weeks after six months. The best response will be evaluated on the basis of new response evaluation criteria in solid tumors (Revised RECIST guideline [version 1.1]). AEs defined by the National Cancer Institute's Common Terminology Criteria for Adverse Events (version 4.0) will be assessed during the study treatment period.

### Statistical analysis

The primary endpoint of this study is the ORR. The protocol regimen in this study would be the fourth‐generation or later treatment regimen for most of the enrolled patients because the treatment after osimertinib is the same as that used in patients without driver oncogene mutations.^10^ The threshold overall response rate has been set at a lower level than that used in prior chemotherapy treatment for this population, and it is almost the same as that used in salvage therapy with EGFR‐TKI for patients with an acquired resistance to prior EGFR‐TKI. With regard to the prior chemotherapy, in an EAST‐LC trial, a randomized phase III study comparing the efficacy of S‐1 with docetaxel as a second‐line treatment for patients whose driver oncogene mutations were unknown, the ORRs of S‐1 and docetaxel were 8.3% and 9.9%, respectively.^11^ Concerning the salvage chemotherapy with EGFR‐TKI, the response rate of treatment with erlotinib after acquired resistance against gefitinib was 4.3%.^12^ Based on these findings, the threshold ORR has been set to 4% in this study because eligible participants have been first treated with osimertinib, a third‐generation EGFR‐TKI. In addition, the ORR of afatinib and bevacizumab combination therapy, following treatment with a first‐generation EGFR‐TKI, was 19%, which was considered the predicted ORR.^13^ Based on this assumption, the number of patients satisfying 80% power for one side with a significance level of 5% was 35. Finally, 37 cases were set as the sample size in this study in consideration of the possibility of unqualified cases. JMP 12 software (SAS Institute, Inc., Cary, North Carolina) will be used for statistical analysis.

## Discussion

This prospective phase II multicenter, open‐label, single‐arm trial aims to evaluate the efficacy and safety of afatinib and bevacizumab used as combination therapy for patients with NSCLC with mutations in the *EGFR* gene previously treated with osimertinib. To date, the standard regimen proposed for patients who acquired resistance after osimertinib treatment is similar to the treatment used for patients without any mutations. Generally, the treatment with cytotoxic agents with or without an immune checkpoint inhibitor is less effective and more toxic in these patients than the previous EGFR targeting treatment. Therefore, a better treatment option that includes EGFR‐TKI is needed for these patients.

Although the efficacy of the repeated use of EGFR‐TKI on patients with acquired resistance is not established, clinical data has revealed that this approach might be suitable in some populations.^14^, ^15^ Several reports revealed that afatinib, an irreversible pan‐ErbB family blocker, has potential activity against osimertinib‐resistant lung cancer because it has been proven effective against tumors with both minor and compound *EGFR* mutations. During in vitro cell proliferation assays, afatinib exhibited a lower half maximal inhibitory concentration (IC_50_) than other TKIs, including osimertinib, against lung cancer cell lines with minor *EGFR* mutations.

Bevacizumab, a VEGF‐A‐neutralizing antibody, inhibits neovascularization for cancer growth and improves the tumor microenvironment. Bevacizumab improved the delivery of anticancer drugs to cancer sites as observed in the mouse model by modulating tumor vessel physiology.^16^ Moreover, T790M resistance was overcome through dual blockade of the EGFR and VEGF pathways, thereby inhibiting the cross‐talk between them.^17^, ^18^ Therefore, we hypothesized that, in combination with chemotherapy, salvage treatment with afatinib and bevacizumab will be effective for patients who have previously undergone osimertinib therapy.

In terms of combination therapy that includes afatinib and bevacizumab, some trials were performed to assess patients with *EGFR* mutations in the first‐line setting. During phase I in one particular trial, optimal dosages of afatinib and bevacizumab combination therapy were determined, and it was concluded that a 30 mg dose of afatinib daily and a 15 mg/kg intravenous infusion of bevacizumab administered once every three weeks were well tolerated.^19^ The study then entered a randomized phase II trial to compare this combination therapy with a 40 mg dose of afatinib monotherapy daily in the first‐line setting.^20^ In the trial proposed in this protocol, we decided to conduct a phase II trial with this combination therapy using this recommended dose for patients who have previously received osimertinib treatment.

In conclusion, we believe that the proposed afatinib plus bevacizumab regimen after treatment with osimertinib trial will provide clinically significant data that will aid the development of novel treatment strategies for patients with *EGFR*‐mutated lung cancer who develop therapy resistance after first‐line treatment with osimertinib.

## Disclosure

N.K. received an honorarium and research grant from Chugai Pharmaceutical Co., Ltd. and Boehringer Ingelheim Japan. T.K. received an honorarium and research grant from Chugai Pharmaceutical Co., Ltd. and Boehringer Ingelheim Japan.
